# Journey to the Center of the Cell: Cytoplasmic and Nuclear Actin in Immune Cell Functions

**DOI:** 10.3389/fcell.2021.682294

**Published:** 2021-08-05

**Authors:** Julien Record, Mezida B. Saeed, Tomas Venit, Piergiorgio Percipalle, Lisa S. Westerberg

**Affiliations:** ^1^Department of Microbiology, Tumor and Cell Biology, Karolinska Institute, Stockholm, Sweden; ^2^Science Division, Biology Program, New York University Abu Dhabi (NYUAD), Abu Dhabi, United Arab Emirates; ^3^Department of Molecular Biosciences, The Wenner-Gren Institute, Stockholm University, Stockholm, Sweden

**Keywords:** immune cells, actin, nucleus, mechanosensing, cytoplasm

## Abstract

Actin cytoskeletal dynamics drive cellular shape changes, linking numerous cell functions to physiological and pathological cues. Mutations in actin regulators that are differentially expressed or enriched in immune cells cause severe human diseases known as primary immunodeficiencies underscoring the importance of efficienct actin remodeling in immune cell homeostasis. Here we discuss recent findings on how immune cells sense the mechanical properties of their environement. Moreover, while the organization and biochemical regulation of cytoplasmic actin have been extensively studied, nuclear actin reorganization is a rapidly emerging field that has only begun to be explored in immune cells. Based on the critical and multifaceted contributions of cytoplasmic actin in immune cell functionality, nuclear actin regulation is anticipated to have a large impact on our understanding of immune cell development and functionality.

## Introduction

The immune system is tasked with detecting and responding to harmful non-self and self- molecules through the cooperative action of different cells. Immune cells are among the most motile cells in the body which enables the rapid communication between leukocytes specialized in gathering information about pathogens and altered self with other leukocytes specialized in mounting effector responses in lymphoid organs as well as the delivery of these cells to the sites of inflammation. Thus, leukocytes continually change shape to adapt to their environment but also to communicate with other cells. For instance, at sites of infection chemokine and selectin receptors on the surface of circulating immune cells are engaged by factors released from the endothelial cells lining the capillaries. This causes leukocytes such as neutrophils and lymphocytes to roll on the activated endothelium and trigger outside-in activation of integrin molecules which stimulates the immune cells to slow down and stop (Vestweber, 2015). The immune cell will then polarize to acquire a motile shape, characterized in the direction of movement by the dynamic branched actin network of the lamellipodia and an acto-myosin rich trailing uropod that generates force to propel the cell body forward. This will allow migration on and through the endothelium to reach the inflamed tissue. Endothelial transmigration is often limited by the deformability of the cells’ largest organelle – the nucleus (Vargas et al., 2017). Motility within the inflamed tissue is essential to reach and clear the infection but also in improving the odds of antigen specific immune responses. Immune cells need to directly communicate with other immune cells and diseased cells, and they do so by establishing an immune synapse characterized by a drastic remodeling of their cytoskeleton. While stable in relation to the communicating cells, immune synapses are extremely dynamic, and sites of multiple cellular processes (Dustin, 2014). Studies of primary immunodeficiencies where patients have a mutation in specific actin regulators have provided remarkable insights into the unique and redundant functions of these regulators in immune cells (Saeed et al., 2020).

Defense against non-self pathogens is intuitively seek and destroy. Paradoxically, however, this same detection machinery is used to distinguish harmless non-self pathogens such as commensal bacteria leading to tolerance. Recent studies of have established the importance of immune cells’ ability to sense the mechanical properties of their environment and a role for such mechanosenstion in fine-tuning the strength and outcome of immune cells’ interactions with the environment and between cells (Zhu et al., 2019). Moreover, a growing body of work on nuclear actin dynamics and regulators, mostly in non-immune cells, highlights their role in cell signaling, differentiation, proliferation, and gene transcription. Therefore, these aspects of regulation could play critical roles in shaping the overall outcome of immune responses.

**FIGURE 1:**
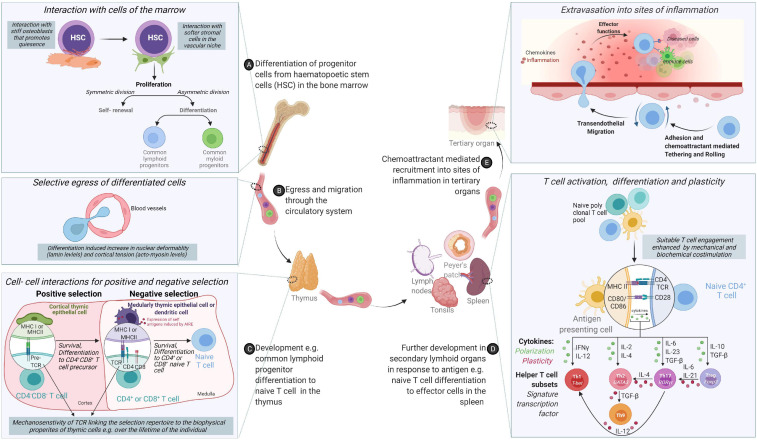
*From origin to function – an odyssey to the battlefront guided through cooperation and tested by competition.***(A)** Cells of the immune system originate from hematopoietic stem cells (HSCs) in the bone marrow via differentiation cues received from the mechanical and chemical composition of the bone marrow. **(B)** Following differentiation, acquisition of migratory behavior enables cells to egress the bone marrow and circulate through the body via lymphatic and circulatory systems. **(C)** While some cells of the innate immune system compartment complete their differentiation in the bone marrow, adaptive cells such as T cells need comprehensive training in self- non-self-discrimination before acquisition of effector molecules through positive and negative selection in secondary lymphoid organs. **(D)** Differentiated immune cells colonize different organs in the body or continue circulating. **(E)** Functional immune cells are called upon to sites of inflammation to control infection or determine the magnitude and nature of immune responses.

Immune cells uniquely face the challenge of requiring a single cell or its lineage to have plastic differentiation potential, migrate and adapt to diverse environments (Figure 1). Here, we describe how the actin cytoskeleton enables sensing of mechanical stimuli at the plasma membrane and its’ transmission to the nucleus. We will also discuss, largely based on studies of non-immune cells, the emerging role of nuclear actin in genomic organization and nuclear functions and discuss the implications such regulation may have on immune cells.

## Setting the Stage: Immune Dysregulation Caused by Mutations in Actin Regulators

The first described so called “actinopathy” is Wiskott–Aldrich syndrome (WAS) in which patients suffer from severe immunodeficiency caused by loss-of-function mutations in the hematopoietic specific WAS protein (WASp). WASp gave the name to the WASp family of actin promoting factors also containing the ubiquitously expressed neural WASp (N-WASp), the WASp-family verprolin-homologous protein/suppressor of the cyclic AMP receptor (WAVE/SCAR), WASp and SCAR homolog (WASH), and WASp homolog associated with actin, membranes, and microtubules (WHAMM) (Campellone and Welch, 2010; Moulding et al., 2013; Alekhina et al., 2017). The activity of WASp family members is regulated by the small Rho GTPases Cdc42 and Rac1/2. WASp family members have a carboxy terminal verprolin cofilin acidic (VCA) domain that mediates binding to the actin related protein (Arp2/3) complex that polymerize actin branching from existing actin filaments (Campellone and Welch, 2010; Moulding et al., 2013; Alekhina et al., 2017). With increased accessibility to whole genome sequencing approaches for immunodeficient patients, patients have been described with mutations in hematopoietic protein-1 (Hem1, a component of the WAVE regulatory complex), Arp2/3 subunit Arp complex 1B (ARPC1B), Cdc42, Rac2, RhoA, and dedicator of cytokinesis 8 [Dock8, a guanine exchange factor (GEF) for Cdc42 and Rac1/2] (Saeed et al., 2020). Moreover, mutations in β-actin and the actin sensor Myocardin Related Transcription Factor A (MRTF-A)/Megakaryoblastic leukemia 1 protein (MKL1) lead to severe immunodeficiencies with poorly functional immune cells (Saeed et al., 2020). As a testimony to the enormous need for rapid cell cytoskeletal adaptation, cells of the hematopoietic lineage often express multiple copies of seemingly redundant regulators of the actin cytoskeleton. This is exemplified by co-expression of hematopoietic-specific WASp and the highly homologous and ubiquitously expressed N-WASp (Cotta-de-Almeida et al., 2007; Recher et al., 2012; Westerberg et al., 2012). Moreover, naturally occurring gain-of-function mutations, such as the WASp^L270P^ and WASp^I294T^ and increased activity of MRTF-A/MKL1 have further increased the understanding of actin regulators in the hematopoietic lineage cells (Devriendt et al., 2001; Ancliff et al., 2006; Keszei et al., 2018; Record et al., 2020). Studies of immune cells from patients and animal models with mutations in actin regulators has revealed the critical role of actin remodeling and dynamics in immune cell functions opening up the question — what are the unique and redundant roles of these regulators in mechanical and biochemical signal transduction from the cell surface to the nucleus?

## Mechanosensing Transmitted to Immune Cell Activation

Cells are exposed to normative and shear mechanical forces from their microenvironment. The complex interplay between such mechanical stresses and cellular behavior were brought to the forefront by pioneering observations that compliance or rigidity of a substrate that presents collagen affects the adherence and motility of differentiated epithelial and fibroblastic cells (Pelham and Wang, 1997) and mesenchymal stem cell differentiation (Engler et al., 2006). In general, structures at the cellular level (microscale) and at the molecular level (nanoscale) that are sensitive to mechanical stimuli enable cellular responses such as activation, differentiation, and migration through their coupling to the cell’s cytoskeleton (Orr et al., 2006). A useful framework for understanding cellular mechanosensing breaks down the process to four distinctive components [reviewed in Chen et al. (2017)]: (1) *mechanopresentation*- the mechanical stimuli is presented to the cells, (2) *mechanoreception*- the ligand is engaged by the mechanoreceptor at the cell surface, (3) *mechanotransmission*- the signal propagates away from the ligand/receptor binding site and, (4) *mechanotransduction*- the mechanical stimuli is converted into a biochemical signal.

## Mechanopresentation

### Stiffness in the Immunological Synapse Sets the Activation Threshold

**FIGURE 2:**
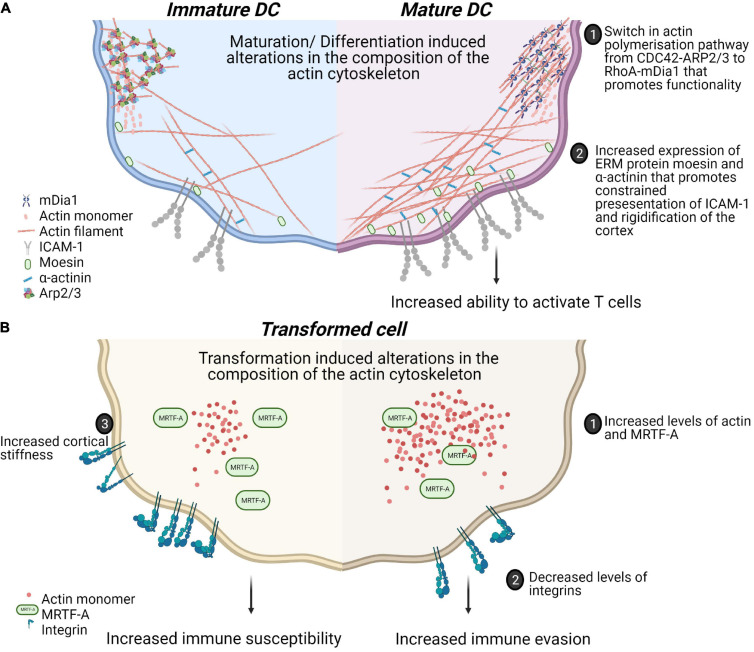
*Physiological and pathophysiological alterations in mechanical properties of immune cells alter functionality of cell-cell contacts.***(A)** Maturation of dendritic cells in response to Th1 polarizing antigens is associated with alterations in actin polymerization pathways. This is associated with a switch from more adherent, micropinocytosis favoring phenotype to a more migratory phenotype. These changes also include increased confinement of membrane proteins and rigidifying of the cotext that collectively enhance the activation of T cells. **(B)** Transformation induces changes in biophysical properties of cells through alterations in the actin cytoskeleton. Increased levels of MRTF contribute to prometastatic changes such as decreased expression of integrins and increased actin content enhancing migration. However, these adaptations could also lead to increased cortical rigidity and increased susceptibility to cytotoxic cells.

Tissue composition and architecture are increasingly implicated in driving inflammation and pathophysiology (Winkler et al., 2020). How the mechanical property, particularly its stiffness or deformability, affects immune cell activation and differentiation have been studied using 2 dimensional hydrogels of varying stiffness in different immune cells [reviewed in Rossy et al. (2018)]. For T cells, the presentation of similar amounts of ligands for the T cell receptor (TCR) and the integrin lymphocyte function-associated antigen 1(LFA-1) on hydrogels with varying stiffness (0.5–100 kPa) showed hydrogel stiffness dependent modulation of cell migration and morphological changes. Interestingly, metabolic properties and cell cycle progression were affected only at the highest stiffness tested (100 kPa) (Saitakis et al., 2017). This is consistent with other reports of maximal cytokine production and proliferation through TCR engagement on stiff substrates (100 kPa) (Judokusumo et al., 2012; O’Connor et al., 2012). Dissecting the specific contributions of the TCR and LFA-1 to spreading on varying stiffnesses revealed a biphasic response when only TCR is ligated and a monotonous response upon LFA-1 co-ligation (Wahl et al., 2019). These results suggest that T cells discriminate between the wide range of substratum stiffnesses found in the body and adapt their responses accordingly. In human dendritic cells (DCs) the culture substrate stiffness during *in vitro* differentiation has been shown to negatively regulate the expression of C-type lectins, β2 integrins as well as podosome formation leading to decreased antigen uptake (Mennens et al., 2017). In addition to the relevance of such studies for *ex vivo* culture and expansion of immune cells, these studies reveal cellular rigidity as an additional layer for regulating cell-cell interactions. In this regard, the rigidity of antigen presenting DCs has been studied in physiological and pathophysiological contexts (Figure 2). Firstly, DCs are particularly potent primers of T cell responses. The DC-T cell immunological synapse is characterized by actin rearrangements in both cells. At the DC side, actin rearrangements through WASp and Hem1 containing WAVE complexes promote the appearance of podosomes, which is thought to restrict the mobility of proteins and increased rigidity that promotes stability of contacts and CD4 T cell activation (Malinova et al., 2016; Leithner et al., 2021). Secondly, during T helper (Th) 1 immune response driving maturation, DCs upregulate levels of the actin cytoskeletal regulators moesin and α-actinin 1 which serves to restrict the mobility of Intercellular Adhesion Molecule 1 (ICAM-1) and promote T cell activation (Comrie et al., 2015). Atomic force microscopy experiments established similar twofold stiffening during *in vivo* LPS mediated DC maturation and *in vitro* differentiation of bone marrow derived DCs (BMDCs). Using BMDCs from murine models of WASp, Fascin or HS1 deficiency, a role for WASp expression in this maturation response was also shown. Surprisingly, this study revealed that DC stiffness lowers the activation threshold for CD4 but not CD8 T cells (Blumenthal et al., 2020). The rigidity of follicular DCs, found in germinal centers, was found to be twofold higher than BMDCs (Spillane and Tolar, 2016). As B cells are thought to preferentially use mechanical extraction of antigens as opposed to enzymatic liberation, tethering of antigens to stiff surfaces in germinal centers could be a critical mechanism for stringent B cell affinity discrimination further expanding the role played by such cell mechanics in immunity.

### Moving Against the Flow

Immune cells circulating in the periphery are exposed to mechanical stress from the blood flow. The recruitment of immune cells during inflammation is regulated by a multi-step cascade of cell rolling, activation, adhesion, and transmigration through the endothelial cell barrier. After selectin-mediated adhesion, immune cells migrate along the activated vascular endothelium on which ligands, including ICAM-1 and vascular cell adhesion molecule-1 (VCAM-1) are expressed. Hematopoietic stem cells use this route to home and migrate into the bone marrow. *In vitro* experiments where cells are introduced to controlled unidirectional shear flow reveal that interaction with VCAM-1 leads to migration downstream of the flow direction whereas LFA-1 – ICAM-1 interactions mediated upstream migration, in reverse direction of the flow (Buffone et al., 2018). Effector T cells oriented their migration on ICAM-1, but not on ICAM-2 and VCAM-1, on endothelial monolayers (Valignat et al., 2013; Dominguez et al., 2015). Neutrophils express LFA-1 and they also express macrophage-1 antigen (Mac-1) that binds to ICAM-1. Neutrophils use Mac-1 to migrate downstream on ICAM-1 coated surfaces or activated endothelium during shear flow. However, blocking of Mac-1 lead to upstream migration through LFA-1 - ICAM-1 interactions (Buffone et al., 2019). Marginal zone B cells that depend on strong adhesion forces to localize to the marginal sinus of the spleen where they are exposed to shear stress from the blood flow show similar differential migration in response to VLA-1 and LFA-1 (Tedford et al., 2017). This shows that adhesion molecules steer migration under shear flow stress and that LFA-1 is a key receptor responsible for upstream migration on the endothelium for transmigration of various immune cells into tissues.

## Mechanoreception and Mechanotransmission

### Immune Receptors Act as Mechanosensors

Mechanoreception by the cell is mediated by cell surface receptors and ion channels and transmitted from these receptors by force induced conformational changes or alteration of receptor-ligand bond properties [reviewed in Zhu et al. (2019)]. While the responsiveness of integrins and ion channels to mechanical force have been appreciated for some time, the ability of immunoreceptors such as the T cell receptor (TCR), B cell receptor (BCR), FcγRIIA (the receptor for the Fc fragment of immunoglobulin G, IgG) and glycoprotein Ib (GPIb) to directly sense force was surprising. Direct mechanoreception was initially demonstrated for the TCR where controlled presentation of mechanical stimulus led to T cell activation regardless if the force is tangential or normal to the receptor (Kim et al., 2009; Li et al., 2010; Pryshchep et al., 2014; Feng et al., 2017). Further characterization of the mechanosensing capacity of the TCR showed that the TCR can generate forces in the 12–19 pN range during binding with peptide-MHC molecules. While weak compared to forces generated by integrin-ligand bonds, these forces contribute to the remarkable sensitivity of TCR triggering and affinity discrimination (Das et al., 2015; Liu et al., 2016). Recently, it was shown that selection of CD8 T cell in the thymus relies on a mechanotransduction signaling loop which via TCR–pMHC–CD8 heterotrimer enables cells to differentiate signals for positive or negative selection (Hong et al., 2018). The TCR acts as a mechanosensory receptor in the early steps of T cell activation and is dependent on the actin cytoskeleton and can also be modulated by co-engagement of the integrin LFA-1 (Husson et al., 2011). Mechanical activation of the TCR leads to the generation of cytoskeletal forces from actin polymerization and myosin mediated retrograde actin flow enabling the cell to counteract mechanical forces exerted on the TCR (Bashour et al., 2014; Hui et al., 2014; Colin-York et al., 2019). Thus, actin cytoskeletal dynamics enable crosstalk between the cell and its environment (Gupta et al., 2015) and critically modulate T cell activation thresholds (Kaizuka et al., 2007; Babich et al., 2012; Murugesan et al., 2016; Jankowska et al., 2018). Intriguingly, while abrogation of actin cytoskeletal dynamics prevents T cell activation through mechanical stimulation, applying oscillatory forces on T cells with an inhibited actin cytoskeleton still leads to cellular activation. This suggests the formation of short-lived signal intermediates that accumulate to successfully activate the T cell if the force that trigger the TCR is applied with a high frequency (Hu and Butte, 2016). The development of sensitive probes such as double strand DNA tension sensors, revealed differential sensitivity of the IgM or isotype switched IgG/IgE B cell receptors (BCRs) (Wan et al., 2015).

## Mechanotransduction

### Rho GTPases as Messengers in Immune Cells

Mechanotransduction is the translation of mechanical stimuli sensed by the mechanoreceptor into biochemical signals which activates signaling pathways inside the cell via cytoskeletal remodeling. In a wide range of cells, the Ras homology gene family, member A (RhoA) pathway is activated in response to forces particularly downstream of integrin signalling and has been extensively studied in this context as it regulates both actin cytoskeletal changes and myosin contractility. When tension is applied, for instance, on α5β1 integrins, it promotes the binding of the fibronectin synergy motif leading to the activation of focal adhesion kinase (FAK) (Friedland et al., 2009) and the Src family of kinases (SFKs) (Na et al., 2008). Activation of SFK and FAK leads to the activation of the RhoA GEFs LARG and GEF-H1, respectively (Guilluy et al., 2011). Rho GTPases act as molecular switches alternating between their activated GTP-bound form and their inactivated GDP-bound form. GTP-bound active RhoA regulates both actin polymerization by direct activation of the formin mDia1 (Watanabe et al., 1999) as well as cell contractility via Rho-associated protein kinase (ROCK) mediated phosphorylation of the regulatory light chain of myosin (MLC). ROCK can directly phosphorylate MLC on Serin-19 (Amano et al., 1996) and inhibit the MLC phosphatase MYPT (Kimura et al., 1996). RhoA further promotes buildup and reorganization of actin filaments by phosphorylating and activating LIM kinase which in turn phosphorylates cofilin and prevents its filament severing activities (Maekawa et al., 1999).

### Deficiency in Rho GTPases Cause Immune Cell Alterations

Dysregulation of Rho GTPases is associated with cell transformation and immunodeficiency (Hall, 2012). RhoA is important for immune cell functions. In B cells, RhoA regulates BCR signaling with the loss of RhoA activity abolishing calcium influx and proliferation upon BCR activation (Saci and Carpenter, 2005). RhoA is also activated through p190RhoGEF upon CD40 stimulation and drives plasma cell differentiation (Lee et al., 2003; Ha et al., 2012). RhoA regulates T cell development and function with T cells devoid of RhoA showing a block at the pre-TCR stage during thymic development (Zhang et al., 2014). Cytokinesis and chromosome segregation is impaired when the RhoA GTPase activating protein (GAP) ARHGAP19 is overexpressed in T cells (David et al., 2014). Activation of integrins is regulated by RhoA in T cells (Smith et al., 2003; Vielkind et al., 2005) controlling T cell polarization with the formation of the leading edge and the uropod necessary for transendothelial migration (Heasman et al., 2010). Furthermore, T cell migration in non-lymphoid tissues is dependent on RhoA and the absence of RhoA in these cells results in impaired control of skin infections (Moalli et al., 2018). Finally, RhoA regulates immune synapse formation by adjusting cofilin activity. The increased expression of RhoA in naïve T cells leads to the formation of smaller and stiffer immunological synapses when compared to synapses formed by effector T cells which have a relatively lower levels of RhoA (Thauland et al., 2017). Another well know role of RhoA, is the activation of the MKL1/MRTF-A/serum response factor (SRF) pathway which downstream of actin changes regulates numerous genes including cytoskeletal genes. This highlights the importance of RhoA in the coordination of mechanical cues and cellular response to these stimuli via the actin cytoskeleton.

## Cytoplasmic Communication to the Nucleus

**FIGURE 3:**
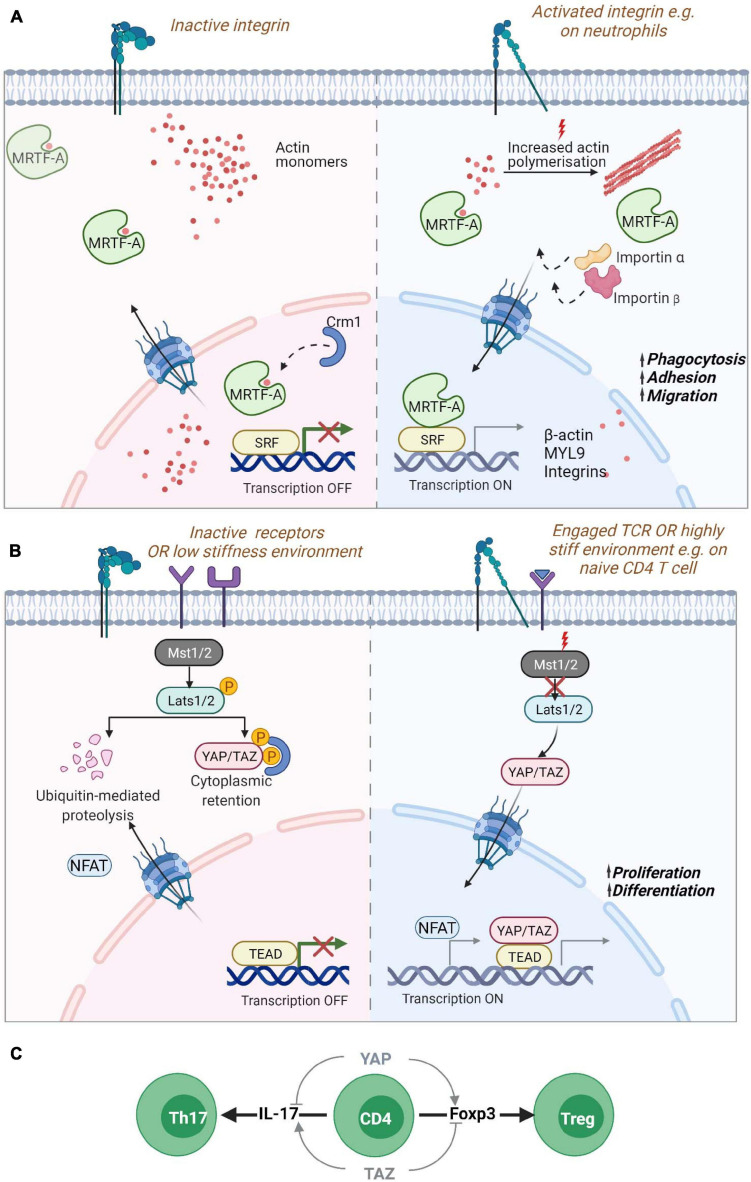
*Nucleo-cytoplasmic shuttling proteins.* Mechanical information from the cell surface can be transduced to the nucleus through the shuttling of gene regulators such as **(A)** MRTF-A/MKL1 or **(B)** YAP or TAZ. **(A)** MRTF-A contains two RPEL domains that can bind to monomeric actin sequestering them in the cytoplasm. Reduction of actin monomers due to filament build up in the cytoplasm leads to the exposure of NLS sequences in MRTF-A, promoting its transport via the chaperones importin α/β into the nucleus. Inside the nucleus, reduced levels of monomeric actin enable its binding to SRF transcription factors for the expression of cytoskeletal genes. Increased levels of monomeric actin in the nucleus in turn promote its association with Crm1 chaperone and export from the nucleus. MRTF-A nuclear translocation is also regulated by phosphorylation (not shown). In neutrophils, MRTF-A is known to drive the expression of β-actin, myosin light chain (MLC) and integrins promoting phagocytosis, adhesion and migration. **(B)** The YAP/TAZ proteins are retained in the cytoplasm via phosphorylation through activated Lats1/2 kinase targeting the proteins for degradation or retention. Mechanosensitive signaling leading to reduced phosphorylation of Lats1/2 by Mst1/2 leads to hypo-phosphorylation of the protein and its translocation to the nucleus for association with TEAD containing transcriptional regulators. YAP regulates the enhanced proliferation of T cells on stiff environments. **(C)** During naïve T cell differentiation, YAP and TAZ have opposing effects. The expression of the YAP paralog promotes Foxp3 expression and thus, regulatory T cell formation while also inhibiting IL-17 expression Th17 differentiation. While TAZ promotes Th17 differentiation and blocks Treg differentiation. Myocardin Related Transcription Factor A (MRTF-A)/Megakaryoblastic leukemia 1 protein (MKL1); Yes-associated protein (YAP) and its paralog WW Domain-Containing Transcription Regulator Protein1 (WWTR1/TAZ); Nuclear localization signal (NLS); Serum response factor (SRF); TEA domain family (TEAD).

The nucleus can sense mechanical stimuli from the environment through biochemical signal transduction from mechanoresponsive cell surface receptors resulting in nucleo-cytoplasmic shuttling of mechanoresponsive biochemical cues (Figure 3) or more directly, through coupling of cytoskeletal proteins and motors with mechanoresponsive elements in the nuclear envelop and nucleoplasm (Cho et al., 2017; Pennacchio et al., 2021).

## Nucleo-Cytoplasmic Shuttling Proteins

### Myocardin Related Transcription Factor A (MRTF-A)/Megakaryoblastic Leukemia 1 Protein (MKL1)

The Myocardin Related Transcription Factor A (MRTF-A), also known as Megakaryoblastic leukemia 1 protein (MKL1) (Ma et al., 2001), is probably the best described mediator between cytoskeletal dynamics and nuclear responses. MRTF-A is a transcription cofactor that binds to SRF to regulate expression of numerous genes and is involved in several processes such as development, cell migration, and immunity (Arsenian et al., 1998; Cen et al., 2004; Esnault et al., 2014; Record et al., 2015). MRTF-A binds to G-actin via its RPEL domain which can form tri- and pentameric complexes with G-actin, serving as a sensor for the levels of G-actin monomers inside the cytosol (Miralles et al., 2003; Vartiainen et al., 2007; Mouilleron et al., 2008; Mouilleron et al., 2011). A second RPEL domain contains an extended bipartite nuclear localization signal (NLS) that is hidden when MRTF-A is bound to G-actin. In the absence of interaction with G-actin, the MRTF-A NLS can interact with importin αβ and promote nuclear translocation of MRTF-A (Mouilleron et al., 2008; Pawłowski et al., 2010; Hirano and Matsuura, 2011; Panayiotou et al., 2016). Activation of RhoA dependent actin polymerization by mechanical or mitogenic stimuli leads to a decrease in actin monomer concentration releasing MRTF-A and promoting its nuclear import via its exposed NLS (Cen et al., 2004; Zhao et al., 2007; Muehlich et al., 2008; Esnault et al., 2014). Therefore, MRTF-A constantly shuttles between the cytoplasm and the nucleus depending on its interaction with actin monomers. MRTF-A/G-actin interactions in the nucleus on the other hand leads to MRTF-A nuclear export via interaction with the nuclear export chaperone Crm1 (Miralles et al., 2003; Vartiainen et al., 2007). In the nucleus MRTF-A binds to SRF and acts as a co-transcription factor when its actin binding is disrupted. Nuclear formin mediated actin polymerization via and depolymerization by MICAL2 (Molecules Interacting with CasL 2) have been shown to be an important regulator of MRTF-A/SRF (Baarlink et al., 2013; Lundquist et al., 2014; McNeill et al., 2020). Phosphorylation of MRTF-A also regulates its localization in cells with phosphorylation of residue Serin-549 by ERK resulting in MRTF-A export from the nucleus (Miralles et al., 2003; Muehlich et al., 2008). Other phosphorylation sites also regulate MRTF-A localization such as residue Serin-98 that when phosphorylated prevent the interaction between MRTF-A and G-actin leading to MRTF-A nuclear accumulation. Phosphorylation of Serin-33 increases the activity of a Crm1-dependent nuclear export signal (NES) (Panayiotou et al., 2016). However, the mechanisms regulating these phosphorylation events are unknown.

A homozygous nonsense mutation p.K723X in MRTF-A leading to a strongly reduced expression of the protein has been described in a patient presenting with severe immunodeficiency (Record et al., 2015). The patient suffered from recurrent and severe bacterial infections, such as Pseudomonas septic shock associated with meningitis, malignant otitis externa and abundant cutaneous and subcutaneous abscesses suggesting impaired innate immune cell functionality. This was confirmed by reduced levels of monomeric and filamentous actin in neutrophils which showed severe defects in phagocytosis and migration. MRTF-A deficient cells displayed a decrease in the expression of MYL9 but increased expression of the integrin CD11b which could explain the uropod retraction defect and the failed migration (Record et al., 2015) (Figure 3A). More recently, two siblings were reported with a frameshift mutation p.V453Rfs^∗^16 resulting in the absence of MRTF-A in patient cells and severe migratory defects in patient’s neutrophils (Sprenkeler et al., 2020).

### Yes-Associated Protein (YAP) and Its Paralog WW Domain-Containing Transcription Regulator Protein1 (WWTR1/TAZ)

Another important pathway for mechanotransduction into the nucleus is the Hippo pathway that activates transcription by Yes-associated protein (YAP) and its paralog WW Domain-Containing Transcription Regulator Protein1 (WWTR1/TAZ) proteins (Oka and Sudol, 2009). YAP and TAZ are both considered as mechanotransducers that relay signals about substrate stiffness during cell spreading and cell-cell interactions (Dupont et al., 2011; Kim et al., 2011; Nardone et al., 2017). When the Hippo pathway is “On,” it leads to the phosphorylation of YAP (on Serin-127) and/or TAZ (Serin-89) via the kinase linker of activation (LAT)-Serin-172 (LATS1/2) resulting in the cytoplasmic sequestration or degradation of these proteins (Meng et al., 2016). The activation of Rho GTPases or of FAK - Phosphoinositide 3-kinase (PI3K) signaling during attachment to the extracellular matrix leads to inhibitory phosphorylation of LATS1/2 resulting in the translocation of YAP/TAZ into the nucleus (Mo et al., 2012; Zhao et al., 2012; Kim and Gumbiner, 2015). The stability of the actin cytoskeleton is essential for YAP shuttling into the nucleus. Disruption of F-actin promotes the phosphorylation of YAP and blocks its nuclear localization (Mo et al., 2012; Zhao et al., 2012). YAP/TAZ shuttling is also regulated by mechanical cues from the extracellular matrix and when cells are exposed to stiff matrices, forces are transmitted from the focal adhesions to the nucleus via the cytoskeleton leading to the stretching of the nuclear pores and increased import of YAP into the nucleus (Elosegui-Artola et al., 2017) (Figure 3B). In the nucleus, YAP/TAZ bind to TEA domain transcription factors (Zhao et al., 2008). YAP/TAZ interactions with TEA activates the transcription of many genes involved in cell growth cell-matrix interactions, extracellular matrix composition and cytoskeleton integrity (Aragona et al., 2013; Morikawa et al., 2015; Zanconato et al., 2015; Nardone et al., 2017).

While there is increasing evidence for the role of the Hippo pathway in immune cells (Yamauchi and Moroishi, 2019), the specific contributions of YAP and TAZ have only recently been unveiled. YAP and TAZ have distinctive roles in T cells displaying reciprocal regulation in the differentiation and function of specific T cell subsets. TAZ regulates T regulatory (Treg) and Th17 differentiation while YAP supports Treg cell functions. In Th17 cells, TAZ co-activates the transcription factor RORγt which is critical for the expression of the cytokine IL-17 and Th17 differentiation. Thus, TAZ enhances Th17 differentiation and potentiates Th17 mediated autoimmune diseases. On the other hand, TAZ attenuates Treg cell differentiation by promoting degradation of the Treg master transcription factor Foxp3 (Geng et al., 2017). YAP seems to have a distinctive role from TAZ and promotes Treg cell differentiation when overexpressed, possibly via TGFβ receptor 2 (Fan et al., 2017). Such a role for YAP in Treg cells has been confirmed in another study showing that YAP sustains Foxp3 and repress IL-17 and IFNγ expression (Figure 3C). YAP promotes expression of genes involved in TGFβ/Smad signaling and suppresses anti-tumor activity leading to increased tumor growth (Ni et al., 2018). Moreover, recent data show that the absence of YAP increases the activity of cytotoxic T cells and leads to higher production of pro inflammatory cytokines, increased expression of Granzyme B and Perforin, and decreased expression of exhaustion receptors, indicating higher tumor killing capacity by YAP deficient T cells (Lebid et al., 2020). Interestingly, it has been shown that T cells proliferate less in soft microenvironments compared to stiff microenvironments. Strikingly, T cells lacking YAP in soft microenvironment proliferate as fast as T cells in stiff microenvironment indicating that stiffness dependent T cell proliferation depends on YAP activity. On soft matrix, YAP is hyperphosphorylated on the Serine 397 and impairs NFAT1 nuclear localization affecting T cell activation and metabolism. As the stiffness of lymph nodes decreases when infection is resolved, YAP activity via mechanosensing of the tissue mechanical properties is speculated to dampen post-infection T cell responses (Meng et al., 2020) (Figure 3B).

## Nuclear Mechanosensing Through Cytoskeletal Coupling

### LINCing the Nuclear and Cytoplasmic Cytoskeletons

**FIGURE 4:**
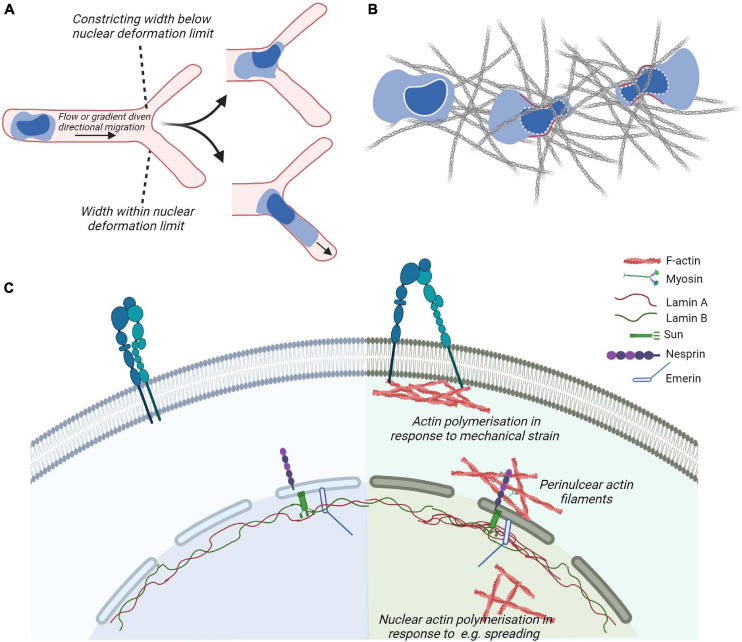
*Cytoskeletal coupling of the nucleus and cytoplasm.***(A,B)** In leukocytes navigating confined environment such as vessel entrances or dense matrices, the mechanical properties and responses of their nuclei play important regulatory roles such that constraints induce nuclear envelope disassembly and generation of forces (in red) to promote leukocyte escape from the compressive environment and identify paths of least resistance. **(C)** Actin cytoskeletal adaptations to plasma membrane proximal signaling or mechanical strain, e.g., through the generation of focal adhesions and perinuclear actin filaments couple to the nuclear cytoskeleton through the nuclear envelop. The outer nuclear envelop proteins such as Nesprin confer recognition of cytoplasmic cytoskeletal proteins such as actin or microtubules (not shown). Inner envelop proteins such as Sun and Emerin enable the connection of Nesprin family of proteins to the intermediate filaments making up the nuclear lamina. The nuclear lamina contribute to the mechanical properties of cell and adapt to mechanical stimulation, e.g., by displaying localized accumulation in response to strain.

Approaches for quantitatively studying the mechanical properties of the nucleus and for determining the relevance of various biomolecules in mechanotransduction have included nuclear deformation in response to controlled presentation of force at the plasma membrane and nuclear positioning during migration (Obino et al., 2016). During migration, the progressive positioning of the nucleus toward the leading edge allows leukocytes to use their largest organelle to sense the path of least resistance for fast migration through tissues (Renkawitz et al., 2019) (Figure 4B). When the migrating cell is subjected to compression that exceeds the deformability of the nucleus, the nuclear envelop disassembles, triggering the generation of resistant pushing forces enabling the cell evade (Lomakin et al., 2020) (Figure 4C). Neutrophils, which have multi-lobed deformable nuclei, or cells with induced nuclear flexibility do not display perinuclear actin accumulation when encountering restrictive spaces, while cells with rigid nuclei do (Thiam et al., 2016). The eukaryotic nuclear envelop is a double membrane bilayer where the with an inner nuclear membrane attached to nucleoskeleton and chromatin and an outer membrane that forms a continuum with the endoplasmic reticulum (ER). Cell nuclei are physically integrated with the cytoskeleton through the mechanosensitive components of the nuclear envelop such as the Linker of Nucleoskeleton and Cytoskeleton (LINC) complexes and the intermediate filaments of the nucleoskeleton that in response to mechanical stresses can undergo physical unfolding revealing cryptic sites and changes in biochemical property (Wong et al., 2021) (Figure 4A). Interestingly, during DC migration, perinuclear actin drives passage through constrictive structures independently of the LINC complex (Thiam et al., 2016).

The LINC complex is composed of outer nuclear envelop membrane protein Nesprin containing the KASH (Klarsicht, ANC-1, Syne Homology) domain. The KASH domain interacts directly with inner nuclear envelop membrane SUN (Sad1p, UNC-84 domain) proteins, that are anchored to the nucleoskeleton by interactions with the carboxyl terminus of Lamin A (Haque et al., 2006; Chang et al., 2015). Various isoforms of Nesprins exist and are coupled to different types of the cytoskeleton as well as motor proteins. These include the largest isoforms of Nesprin-1 and -2 containing an N-terminal actin-binding domain that enables interaction with cytoplasmic actin filaments and signal transduction from actin caps or form transmembrane actin-associated nuclear (TAN) lines. Moreover, these isoforms contain spectrin-repeats that can interact with kinesin and/or dynein subunits and thus microtubules. Nesprin-3 can bind to intermediate filaments via plectin. Nesprin-4, which is only expressed in secretory epithelial cells, is a kinesin-binding protein that connects the nucleus to microtubules. SUN proteins of the inner nuclear envelop are highly evolutionarily conserved. Studies in non-immune cells demonstrate that depletion or expression of dominant-negative Nesprin or SUN proteins severely impairs nucleo–cytoskeletal force transmission (Lombardi et al., 2011). The nuclear lamina is composed of alpha helical rods of type V intermediate filaments that assemble hierarchically to produce a thin meshwork associated with the nuclear side of the inner nuclear membrane. The nuclear lamina is thought to be associated with chromatin through nuclear envelope transmembrane proteins (Wilkie et al., 2011; Solovei et al., 2013; Zuleger et al., 2013). Lamin A/C contribute to nuclear mechanics such that local pressure induces local accumulation of Lamin A (Lammerding et al., 2006). Moreover, in adherent cells, Lamin A/C and transcriptionally active chromatin were shown to be vertically polarized. Perinuclear actin fibers enriched in contractile myosin fibers were linked to the LINC complex and disorganized by inhibition of myosin activity or in laminopathies (Khatau et al., 2009; Kim and Wirtz, 2015). The absence of Lamin A results in a decrease in RhoA activity, reduced adhesion, softened the cytoplasm, and decreased cell migration. This suggests a cross-talk between the nuclear skeleton and the cytoskeleton via RhoA (Hale et al., 2008). Moreover, the proteins of the nuclear envelope Lamin A/C and Emerin regulate MRTF-A translocation into the nucleus. In cells lacking Lamin A/C, actin polymerization is altered resulting in a sequestration of MRTF-A in the cytoplasm. Ectopic expression of Emerin reverted the phenotype (Ho et al., 2013). Emerin stimulation of MRTF-A/SRF activity is dependent on the substrate stiffness (Willer and Carroll, 2017), reinforcing the importance of MRTF-A in mechanotransduction.

### Nuclear Viscosity, Cell Polarization and Migration

Somatic cells express Lamin A/C and Lamin B1 and B2. Genetic disorders that result in altered Lamin A expression or duplication of Lamin B result in human diseases and are collectively termed laminopathies (Rankin and Ellard, 2006). These diseases as well as Lamin A/C and Lamin B deficient mice indicate a tight coupling of Lamin A expression to muscle, nerve and cardiac cell functionality and viability. Mouse embryonic fibroblasts that lacked Lamin A/C or had the truncated form of the gene lacking the CAAX motif had increased numbers of misshapen nuclei, severely reduced nuclear stiffness, and decreased cell viability under strain (Lammerding et al., 2006; Swift et al., 2013). The nucleus is often the largest and stiffest (i.e., most resistant to deformation) organelle in the cell with the nucleoplasm having higher viscoelastic modulus compared to the cytoplasm in various cell types (Noel et al., 2008). Leukocyte nuclei are 50- to 100-fold softer than the nuclei of most non-hematopoietic cells (Shin et al., 2013) due to their low expression of Lamin A/C, a key regulator of nuclear lamina stiffness (Shin et al., 2013). This is thought to facilitate transendothelial migration and during migration in tissues. Although easily deformed, the leukocyte nuclei are nonetheless stiff enough to compress the endothelial cortical cytoskeleton such that it can get displaced and possibly disassembled independently of endothelial myosin IIA contractility (Shin et al., 2013). Transmigration of T cells leads to nuclear lobulation that is independent of the contractility of the underlying endothelium but dependent on myosin IIA to complete the movement of their relatively rigid nucleus through the endothelial junctions (Jacobelli et al., 2013). Moreover, during the activation of naïve T cells with ligand coated beads actin filament accumulation at the interface resulted in nuclear elongation (Gupta et al., 2012; Fabrikant et al., 2013).

Lamin A/C are not expressed in non-activated T cells but transiently upregulated following TCR activation. In adoptive transfer experiments, Lamin A null T cells failed to respond to recall stimulation. Strikingly, Lamin A deficiency led to aberrant immunological synapse formation and altered TCR dynamics and reduced activation (González-Granado et al., 2014). Quantitative proteomic analysis of naïve T cells from wildtype or Lamin A knockout mice stimulated by TCR activation, showed a direct role for Lamin A in regulating expression of epigenetic modifying enzymes. Functional consequence of these changes was reduced ability for T cell polarization to the Th1 T cell subset and enhanced Treg cell differentiation and functionality *in vitro*. The preferred Treg cell differentiation of Lamin A deficient T cells was further confirmed using an *in vivo* model for inflammatory bowel disease in which Lamin A deficient T cells were protected from disease development (Toribio-Fernández et al., 2019). This shows a direct role for Lamin A in controlling differentiation of T cell subsets, critical for T cell mediated immunity.

The centrosome plays an important role for cell polarity and at steady state in B cells, the centrosome is tethered to the nucleus via the LINC complex by centrosome-associated Arp2/3 that locally nucleates F-actin (Obino et al., 2016). Upon B cell activation, Arp2/3 is partially depleted from the centrosome leading to a reduction in F-actin at the centrosome, detachment from the nucleus, and polarization to the immunological synapse. Actin filament density at the centrosome is negatively affected by the degree of cell spreading following BCR and LFA-1 activation (Inoue et al., 2019), suggesting that actin filaments constitute a physical barrier blocking elongation of nascent microtubules. Moreover, during differentiation of hematopoietic stem cells to myeloid lineage cells, large invaginations on the swelling nucleus are generated by microtubule constraints. These invaginations are associated with a local reduction of Lamin B density, local loss of heterochromatin H3K9me3 and H3K27me3 marks, and changes in expression of specific hematopoietic genes. This highlights the role of microtubules in defining the unique lobulated nuclear shape observed in myeloid progenitor cells and suggests that this nuclear shape is important to establish hematopoietic lineage genes (Fabrikant et al., 2013; Biedzinski et al., 2020).

## Nuclear Actin in Transcription, Chromatin Remodeling, and Nuclear Reprogramming

**FIGURE 5:**
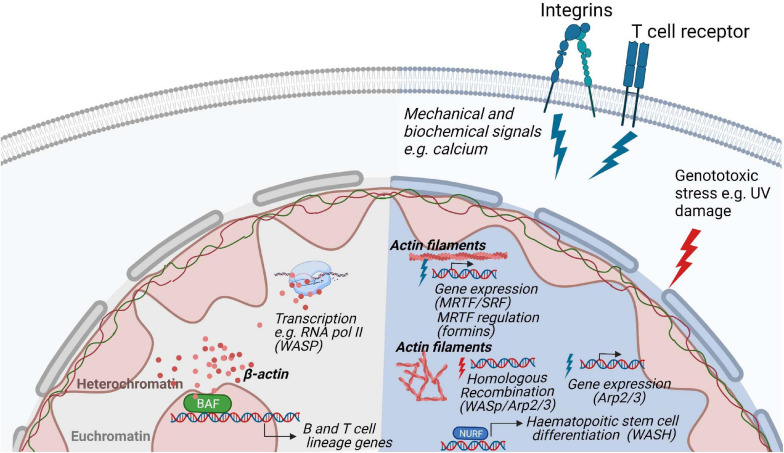
*Regulation of nuclear functions by the actin cytoskeleton.* Nuclear actin plays a role in genome organization and associates and with the transcription machinery. In hematopoietic cells, the actin based chromatin remodeller BAF is involved in the development of B- and T lymphocytes lineages. In response to stimulation, polymerization of actin filaments through various biochemical pathways regulate gene expression and nuclear damage responses. During homologous recombination, WASp, Arp2/3 and actin polymerization promote the movement of DNA damaged foci to the nuclear lamina for repair. In T cells, WASp is involved in RNA Pol II dependent transcription of T cell development genes and it absence results in a decreased of RNA pol II transcription.

Cytoskeletal proteins such as actin, myosin, Arp2/3, and WASp family of proteins have recently emerged as key regulators of nuclear functions [reviewed in Ulferts et al. (2021)]. In eukaryotic cells, they have been implicated in chromatin and transcription regulation and more recently there is evidence for their involvement in chromosomal movements, and functional architecture of the genome (Visa and Percipalle, 2010; Miyamoto and Gurdon, 2013; Percipalle, 2013; Kelpsch and Tootle, 2018). These nuclear actin-based mechanisms appear to impact on key cellular processes such as DNA repair and nuclear reprograming during differentiation (Venit et al., 2020b; Xie et al., 2020) by leveraging on regulated actin dynamics (Plessner and Grosse, 2019) (Figure 5). In hematopoietic cells, WASp family members are in the nucleus and can have activity both dependent and independent on their capacity to induce Arp2/3 mediated actin polymerization.

### Nuclear Actin in Transcription and Chromatin Remodeling

Soon after initial discoveries of actin in the nucleus, its association with RNA Pol II and direct involvement in transcription was revealed (Egly et al., 1984; Scheer et al., 1984; Percipalle et al., 2001, 2003). Actin is associated with all the RNA polymerases facilitating recruitment of histone modifying enzymes (Kukalev et al., 2005; Obrdlik et al., 2008) and forms a complex with Nuclear myosin 1 (NM1), an isoform of the Myo1C gene (Pestic-Dragovich et al., 2000; Fomproix and Percipalle, 2004; Hofmann et al., 2004; Hu et al., 2004; Philimonenko et al., 2004; Grummt, 2006; Dzijak et al., 2012). NM1 can switch between polymerase-bound actin and the ATPase SNF2H, which is a part of chromatin remodeling complex B-WICH. Repositioning of the nucleosomes by B-WICH remodeler leads to the binding of histone acetyltransferase P300/CBP-associated factor (PCAF) and histone methyltransferase Set1 to DNA. This leads to acetylation and methylation of histone H3, generating a more favorable conformation for RNA polymerase to access the chromatin and transcribe genes (Percipalle and Farrants, 2006; Sarshad et al., 2013; Almuzzaini et al., 2015; Almuzzaini et al., 2016; Venit et al., 2020a).

Actin itself is part of several remodeling complexes, with BAF (Brg1- or BRM-associated factors) complex being the most studied (Peterson et al., 1998; Olave et al., 2002; Rando et al., 2002). Out of more than 15 subunits of the BAF complex, the ATPase Brg1 forms a backbone for the whole remodeler and is associated with β-actin (He et al., 2020). This interaction is critical for BAF association with the chromatin as deletion of β-actin leads to dissociation of Brg1 from DNA, an increase of repressive histone marks, and defective localization of heterochromatin (Xie et al., 2018). Reorganization of heterochromatin can be explained by a dual role of the BAF complex on transcriptional regulation and organization of chromatin. Depending on its interactions, BAF can either activate a set of genes by eviction of Polycomb repressive complexes from DNA, or it can repress other genes in complex with transcription repressor REST and repressive remodeling complex NuRD (nucleosome remodeling and deacetylase) (Shimono et al., 2003; Ooi et al., 2006; Yildirim et al., 2011; Kadoch et al., 2017). Therefore, deletion of actin leads to increased association of polycomb repressive complex 2 subunit EZH2 with the chromatin and decreased association of the REST complex which is accompanied by higher-order chromatin switching between heterochromatin and euchromatin (Mahmood et al., 2020). This can have far-reaching consequences for transcriptional reprogramming, differentiation, and cell fate, as Brg1 is required for maintaining pluripotency of stem cells by regulating expression and binding of pluripotency marker Oct4 together with other pluripotency factors Sox2 and Nanog (Ho et al., 2009; Singhal et al., 2014; King and Klose, 2017). Similarly, during direct reprogramming, β-actin knockout cells failed to be properly differentiated to neurons, adipocytes or osteogenic cells due to Brg1 deposition from respective early differentiation genes (Xie et al., 2018; Al-Sayegh et al., 2020; Gjorgjieva et al., 2020). For hematopoietic lineage cells, deletion of Srg3/mBaf155, a scaffold subunit of the BAF complex, causes defects at both the common lymphoid progenitor stage and the transition from pre-pro-B to early pro-B cells due to failures in the expression of B lineage-specific genes, such as Ebf1 (Early B cell factor 1) and IL7ra (IL-7 receptor alpha chain), and their downstream target genes (Choi et al., 2012). Moreover, mice that are deficient in the expression of Brg1 show defects in early B cell development (Choi et al., 2012) (Figure 5).

### Actin and the WASp Family Proteins in the Nucleus

In most of the remodeling complexes, actin serves in its monomeric state, often bound to different actin-related proteins (Shen et al., 2000; Wu et al., 2005; Kapoor et al., 2013; He et al., 2020). Studying the regulation and functions of polymeric actin proved to be technically challenging as actin does not form typical long fibers visible in the cytoplasm but rather short-lived rods and oligomers present under certain conditions such as serum stimulation (Baarlink et al., 2013; Wang et al., 2020), cellular spreading (Plessner et al., 2015), and cellular stress (Munsie et al., 2012; Figard et al., 2019). A breakthrough in the nuclear actin field came from the development of methods and tools to visualize nuclear actin polymerization using NLS tagged actin-specific chromobodies (Plessner et al., 2015) and the 17-amino-acid peptide LifeActGFP for staining of polymeric actin (Riedl et al., 2008). These tools were critical in visualizing nuclear actin dynamics in living cells and to manipulate its levels to distinguish the function of nuclear and cytoplasmic actin. Actin dynamics has been suggested to be important for transcription and transcriptional reprogramming, as actin polymerization-defective mutants inhibit RNA Polymerase (Pol) I transcription (Ye et al., 2008) and several regulators of actin polymerization have been found to be essential for proper assembly and processing of the transcription machinery (Venit et al., 2020b). For example, transcriptional reactivation of the pluripotency gene Oct4 is dependent on the WASp family member WAVE1 and polymeric actin (Miyamoto et al., 2011; Miyamoto and Gurdon, 2013). Similarly, upon serum stimulation, Arp2/3 and N-WASp, known to be responsible for branching and polymerization of actin filaments in the cytoplasm, induce formation of nuclear actin fibers which serves as a scaffold for RNA Pol II clustering around serum-response genes (Yoo et al., 2007; Sadhukhan et al., 2014; Wei et al., 2020). Similar movement along the actin filaments in the nucleus seems to be present in the relocation of chromosome territories upon gene activation (Chuang et al., 2006; Dundr et al., 2007; Wang et al., 2020), DNA damage (Kulashreshtha et al., 2016), and serum starvation (Mehta et al., 2010). During repair of double strand DNA breaks by homologous recombination, movement of single DNA damage foci toward the nuclear lamina favors DNA repair and this movement is dependent on WASp, Arp2/3, and actin polymerization (Chiolo et al., 2011; Tsouroula et al., 2016; Aymard et al., 2017; Caridi et al., 2018; Schrank et al., 2018) (Figure 5).

In haematopoietic stem cells, WASH associates with the nucleosome remodeling factor (NURF) complex in the nucleus where the NURF complex binds to the promoter of the c-Myc gene, necessary for haematopoietic stem cell differentiation (Xia et al., 2014). The nuclear activity of WASH is dependent on nuclear actin polymerization induced by WASH (Xia et al., 2014). In T cells, WASp associates with RNA Pol II for transcription of specific genes for T cell development and T cell lineage commitment (Taylor et al., 2010; Kuznetsov et al., 2017). Deficiency of WASp expression lead to reduced overall RNA Pol II transcription in T cells (Kuznetsov et al., 2017). The absence of WASp results in increased R-loops related double strand breaks causing genomic instability and an imbalance between Th1 and Th2 cells among WAS patient T cells (Sarkar et al., 2018). Both T cells and B cells devoid of WASp show accumulation of R loops, a three-stranded DNA:RNA hybrid structure that can lead to DNA damage by stalling transcription and replication (Wen et al., 2020). In support of a role for the WASp VCA domain for nuclear function, T cells that express constitutively active WASp-I296T, a mutation found in X-linked neutropenia patients, show interaction with a larger number of genes and accumulate target gene products in the nucleus (Kuznetsov et al., 2017). This suggests that constant exposure of the WASp VCA domain leads to increased nuclear WASp activity. A recent study suggests that actin polymerization in the nucleus may be one of the earliest steps in TCR signaling such that nuclear actin polymerization may even precede actin polymerization at the plasma membrane which organizes the immunological synapse (Tsopoulidis et al., 2019). Intriguingly, nuclear actin polymerization was demonstrated to be required to produce specific cytokines upon TCR stimulation (Tsopoulidis et al., 2019). In the nuclear compartment, actin is therefore likely to regulate several nuclear processes by interacting with a plethora of nuclear factors and this might be tightly regulated by actin dynamics.

## The Importance of Nuclear Actin to Avoid Cell Transformation

### Clearance of Actin for Cell Division

The final stage of cell division, cytokinesis, is tightly coordinated during chromosome segregation and dependent on actin polymerization and depolymerization. At the terminal stage of cytokinesis, the daughter cells are connected by a thin cytoplasmic canal, the cytokinetic intercellular bridge, that is eventually cut in a complex process called abscission (Green et al., 2012; Nähse et al., 2017; Pollard, 2017). In response to the abnormal presence of lagging chromatin between dividing cells, an evolutionarily conserved abscission/NoCut checkpoint delays abscission to prevent the formation of binucleated cells (Mendoza et al., 2009). The abscission/NoCut checkpoint induces arrest in cytokinetic cells with chromatin bridges, high intercellular bridge membrane tension, defective assembly of nuclear pore complexes, and DNA replication stress. The abscission checkpoint relies on prolonged activity of the mechanosensor Aurora B activity (Nähse et al., 2017). Successful abscission depends on clearance of microtubules and actin filaments from the intercellular bridge (Addi et al., 2018). Interestingly, the redox state of actin is important and the oxidase MICAL1 directly oxidizes Met44 and Met47 of F-actin into methionine-R-sulfoxides and triggers depolymerization of the actin filaments (Frémont et al., 2017). Oxidation-mediated clearance of F-actin by MICAL1 is counteracted by actin reduction by methionine sulfoxide reductases (MsrBs). When lagging chromatin is present, actin polymerization is essential to stabilize the cytokinetic intercellular bridge. Actin reduction by the MsrBs reductases is a key component of the abscission checkpoint that favors F-actin polymerization and limits tetraploid cells (Bai et al., 2020). Importantly, depletion of MsrBs reductases in Drosophila cells leads to a specific reduction of F-actin in the cytokinetic intercellular bridge without a global effect on the cellular actin cytoskeleton as evident from the occurrence of normal cell spreading (Bai et al., 2020).

### Dysregulated Actin Polymerization and Cell Transformation

The importance of the abscission/NoCut checkpoint becomes apparent during cell transformation. Cancer cell genomes can contain multiple chromosomal rearrangements. During cell transformation to cancer cells, faulty metaphase progression can lead to presence of chromosomes in the cytokinetic intercellular bridge (Tanaka and Watanabe, 2020). Chromosomal bridges arise in late metaphase from end-to-end chromosome fusions after DNA breakage or telomere instability, incomplete DNA replication, or failed resolution of chromosome catenation (two chromatids structurally linked together during replication). Chromosome bridges can persist post miosis in interphase cells, linking the two daughter cells together (Umbreit et al., 2020). Breakage of chromosome bridges requires acto-myosin generated forces and is partly dependent on activation of the LINC complex as shown using inhibitors for the acto-myosin forces and shRNA for LINC complex proteins (Umbreit et al., 2020). Broken bridge chromosomes undergo mitotic DNA damage and frequent missegregation to form micronuclei. These data indicate that a single cell division error can rapidly generate extreme genomic complexity and genetic instability in daughter cells (Umbreit et al., 2020).

Elevated actin polymerization during mitosis is associated with genomic instability and cell transformation. Gain-of-function mutations of WASp in X-linked neutropenia patients lead to increased F-actin that abnormally localize around the mitotic spindle and chromosomes during their alignment and separation, and during anaphase accumulates within the cleavage furrow around the spindle midzone (Moulding et al., 2007). This results in genomic instability as evident in polyploid cells, cells with micronuclei and structural chromosomal aberrations (Moulding et al., 2007, 2012; Westerberg et al., 2010). The mitotic error of cells expressing constitutively active WASp can be prevented by lowering total F-actin using the Arp2/3 inhibitor CK666. Nuclear actin polymerization is linked to functional integrin signaling and components of the LINC complex, suggesting that cellular adhesion and mechanosensing between the cytoplasm and nucleus contribute to nuclear actin dynamics (Chang et al., 2015). However, mitotic nuclear actin filaments can form upon silencing of the nucleoskeletal proteins Emerin or Lamin A/C or upon disruption of the LINC complex, suggesting that these filaments also can form independently of cell spreading and integrin-dependent signaling (Baarlink et al., 2017).

Elevated F-actin in the cytoplasm is associated with increased cell viscosity leading to activation of mechanosensors such as Aurora A and B during mitosis. Aurora A and Aurora B jointly coordinate chromosome segregation and anaphase microtubule dynamics (Hégarat et al., 2011). Aurora A regulates the actin cytoskeleton reorganization during early mitotic stages by phosphorylation of cofilin, thereby preventing the actin depolymerizing function of cofilin (Ritchey and Chakrabarti, 2014). Aurora B regulates chromosome alignment at metaphase and delays abscission in cells with structural defects or chromatin bridges (Liu et al., 2009). Aurora B activity is crucial in sensing mechanical changes to the centromere during chromosome alignment (Liu et al., 2009). Aurora B is interesting since it is a target for hematopoietic malignancies. A selective Aurora B inhibitor induces growth arrest and apoptosis by the accumulation of 4N and 8N DNA content of human acute leukemia cells *in vitro* and *in vivo* (Yang et al., 2007). On the other hand, overexpression of Aurora B can prevent polyploidy in cells that have chemically and genetically induced mitosis defects (Nair et al., 2009) and rescues mitosis defect in cells expressing constitutively active WASp (Moulding et al., 2012). Cells expressing gain-of-function of WASp activity leads to increased cell viscosity as determined by atomic force microscopy and activation of the Aurora B mechanical sensor during mitosis (Moulding et al., 2012). Increased MRTA-A/MKL1 expression is associated with elevated G- and F-actin in patient B cells leading to tetraploidity, increased proliferation, tumor formation, and development of Hodgkin lymphoma (Record et al., 2020). Together, this suggests that increased F-actin content is associated with genetic instability and malignant transformation.

### Actin Polymerization at the Mitotic Exit

During mitotic exit, the nuclear volume of the two daughter cells is expanding in a process dependent on dynamic assembly of nuclear actin filaments enabling chromatin decondensation. Interfering with the polymerization-competent nuclear actin pool by overexpression of the non-polymerizable actin^R62D^-NLS mutant or the actin exporter exportin-6 results in a decreased volume of the daughter cell nuclei as well as an increase in chromatin compaction at mitotic exit (Baarlink et al., 2017). The post mitotic nuclear actin filaments are tightly controlled by the actin-depolymerizing factor cofilin-1 (Baarlink et al., 2017). The nuclear activity of cofilin-1 is regulated by phosphorylation in a cell cycle dependent process with an increase in phosphorylated and thus inactive cofilin-1 during mitotic exit. For adherent cells, adhesion to the extracellular matrix persists during mitosis. The so called ‘reticular adhesions’ are a class of adhesion complexes mediated by the integrin αvβ5 which are formed during interphase, and preserved at cell–extracellular matrix attachment sites throughout cell division (Lock et al., 2018). Depletion of β5 integrins perturbs mitosis by disruption of reticular adhesions. Formation of reticular adhesions maintains cell–extracellular matrix attachment during mitotic rounding and division as a spatial memory transmission between cell generations. Together, these studies reveal that in addition to mechanosensing by the Aurora B kinase, the oxidative state of F-actin in the abscission checkpoint prevents the formation of genetically unstable, tetraploid cancer cells. Rapidly proliferating hematopoietic linage cells, especially B and T cells activated during an immune response, may therefore be particularly vulnerable to increased or decreased F-actin during mitotic exit. Targeted actin reduction and clearance during cytokinesis may be beneficial in general for cancer patients and specifically in cancer therapy for primary immunodeficiency patients with mutations in actin regulators.

## Perspective

The rapid development of biophysical and bioimaging tools to probe cell mechanics has enabled the study of mechanical properties in immune cells [reviewed in Schneider et al. (2021)], greatly expanding our understanding of how mechanical forces can crosstalk with biochemical signals to regulate cell functions. This shift from an exclusively biochemical understanding of signaling reveals the necessity to incorporate the physical status of tissues and cells in our experimental designs to better elucidate physiology and treat pathologies. The role of mechanosensing in immune cells is emerging as a critical signal transduction pathway and strengthens the evidence for the pivotal role of rapid cytoskeletal dynamics in immune cell functions. Moreover, the development of new tools for visualizing actin in the nucleus together with high throughput genomics data reveal that monomeric and filamentous actin exist in the nucleus and have distinct functions (Venit et al., 2020b) reviving the discussion on the form and function of nuclear actin. Many of the actin regulators described in the cytoplasm have been identified in the nucleus and like actin and nuclear myosin, a major function is through interactions with the RNA polymerase machinery. Recent studies uncovered new roles in chromatin remodeling, DNA repair, establishment of chromosomal territories and, in T cells, alteration of chromosome territories following activation (Ioannou et al., 2015). During an immune response, activation of B and T cells leads to changes in transcription, receptor maturation and hyper proliferation of the selected clone as fast as every 6 h. We can easily imagine that defects in nuclear actin would greatly impact such processes as they rely on DNA repair machinery and chromosomal territories.

Understanding immune cell mechanosensing and nuclear actin function is likely to have direct clinical implications. Efforts using whole genome sequencing have revealed an increasing number of diseases caused by mutations in actin regulators. This subgroup of primary immunodeficiency diseases called actinopathies provide a unique opportunity to understand how deficiency in a specific actin regulator affects immune cells (Saeed et al., 2020). The study of hematopoietic specific actin regulators has focused on their cytoplasmic role. As nuclear actin begins to be understood better, the hopes of targeting actin dynamics to influence immune cell activation and differentiation to enhance or dampen the immune response becomes even more tantalizing. Similarly, the cell mechanical response could be used as an output for drug screening to target early immune cell activity. Finally, as forces regulates T cell activation, it is possible that cancer cell stiffness influences their susceptibility to cytotoxic T cells and may be a therapeutic target. In fact, cancer cells need to carefully tune the rigidity of their actin cytoskeleton via MRTF activity to optimize their resistance to cytotoxic attacks when they metastasize (Tello-Lafoz et al., 2021). Alteration of cancer cells physical properties could be used as a clinical means to improve cytotoxic T cell response and might be of use in combinatory treatments with standard chemotherapies, as well as immunotherapies such as checkpoint blockade antibodies (anti-PD1 and anti-CTLA4 antibodies) and treatment with engineered T cells expressing Chimeric Antigen Receptors [reviewed in Li et al. (2020)].

## Author Contributions

JR, MS, and TV wrote the manuscript. JR, MS, TV, PP, and LW edited the manuscript. All authors contributed to the article and approved the submitted version.

## Conflict of Interest

The authors declare that the research was conducted in the absence of any commercial or financial relationships that could be construed as a potential conflict of interest.

## Publisher’s Note

All claims expressed in this article are solely those of the authors and do not necessarily represent those of their affiliated organizations, or those of the publisher, the editors and the reviewers. Any product that may be evaluated in this article, or claim that may be made by its manufacturer, is not guaranteed or endorsed by the publisher.

## Abbreviations

Arp2/3: actin related protein complex 2/3
BCR: B cell receptor
BMDCs: bone marrow derived dendritic cells
CDK: cyclin-dependent kinase
DC: dendritic cell
Dock8: dedicator of cytokinesis 8
ER: endoplasmic reticulum
F-actin: filamentous actin
FAK: focal adhesion kinase
G-actin: globular actin
GAP: GTPase activating protein
GEF: guanine exchange factor
Hem-1: hematopoietic protein 1
ICAM-1: intercellular adhesion molecule 1
JMY: junction-mediating and regulatory
KASH: Klarsicht ANC-1 Syne homology
LAT: linker of activation
LINC: linker of nucleoskeleton and cytoskeleton
LFA-1: lymphocyte function-associated antigen 1
MCM proteins: minichromosome maintenance (MCM) proteins
mDia: mammalian Diaphanous-related
MHC: major histocompatibility (classes I and II)
MICAL: Molecules Interacting with CasL
MLC: myosin light chain
MRTF-A/MKL1: myocardin related transcription factor A/megakaryoblastic leukemia 1
MsrB: methionine sulfoxide reductase
MTOC: microtubule-organizing center
NES: nuclear export signal
NM1: nuclear myosin 1
NLS: nuclear localization signal
NPC: nuclear pore complex
NPF: nucleation promoting factors
NuRD: nucleosome remodeling and deacetylase
NURF: nucleosome remodeling factor
N-WASp: neuronal Wiskott–Aldrich syndrome protein
ORC: origin recognition complex
PCAF: P300/CBP-associated factor
PCNA: proliferating cell nuclear antigen
PID: primary immunodeficiency
PI3K: phosphoinositide 3-kinase
Pol: polymerase
pre-RC: pre-replication complexes
PSGL-1: P-selectin glycoprotein ligand-1
Rho: Ras homolog gene family
RhoA: Ras homolog gene family member A
ROCK: Rho-associated protein kinase
RPA: replication protein A
TCR: T cell receptor
SRF: serum response factor
SUN domain: Sad1p UNC-84 domain
TAN: transmembrane actin-associated nuclear
Th: T helper (cell)
Treg: regulatory T cell
VCA: verprolin cofilin acidic
VCAM-1: vascular cell adhesion molecule 1
VLA-4: very late antigen 4
WASp: Wiskott–Aldrich syndrome protein
WAVE/SCAR: WASp-family verprolin-homologous protein/suppressor of the cyclic AMP receptor
WASH: WASp and SCAR homolog
WHAMM: WASp homolog associated with actin membranes and microtubules
WWTR1/TAZ: WW domain-containing transcription regulator protein 1
YAP: yes-associated protein.
